# Microglia-containing human brain organoids for the study of brain development and pathology

**DOI:** 10.1038/s41380-022-01892-1

**Published:** 2022-12-06

**Authors:** Wendiao Zhang, Jiamei Jiang, Zhenhong Xu, Hongye Yan, Beisha Tang, Chunyu Liu, Chao Chen, Qingtuan Meng

**Affiliations:** 1 The First Affiliated Hospital, Multi-Omics Research Center for Brain Disorders, Hengyang Medical School, University of South China, 421001 Hengyang, Hunan China; 2 Center for Medical Genetics & Hunan Key Laboratory of Medical Genetics, School of Life Sciences, and Department of Psychiatry, The Second Xiangya Hospital, Central South University, 410008 Changsha, Hunan China; 3 The First Affiliated Hospital, Clinical Research Center for Immune-Related Encephalopathy of Hunan Province, Hengyang Medical School, University of South China, 421001 Hengyang, Hunan China; 4 The First Affiliated Hospital, Department of Neurology, Hengyang Medical School, University of South China, 421001 Hengyang, Hunan China; 5 Department of Neurology, Xiangya Hospital, Central South University, 410008 Changsha, Hunan China; 6 Department of Psychiatry, SUNY Upstate Medical University, Syracuse, NY 13210 USA; 7 Hunan Key Laboratory of Animal Models for Human Diseases, Central South University, 410008 Changsha, Hunan China; 8 Hunan Key Laboratory of Molecular Precision Medicine, Central South University, 410008 Changsha, Hunan China

**Keywords:** Psychiatric disorders, Neuroscience, Stem cells

## Abstract

Microglia are resident immune cells in the central nervous system, playing critical roles in brain development and homeostasis. Increasing evidence has implicated microglia dysfunction in the pathogenesis of various brain disorders ranging from psychiatric disorders to neurodegenerative diseases. Using a human cell-based model to illuminate the functional mechanisms of microglia will promote pathological studies and drug development. The recently developed microglia-containing human brain organoids (MC-HBOs), in-vitro three-dimensional cell cultures that recapitulate key features of the human brain, have provided a new avenue to model brain development and pathology. However, MC-HBOs generated from different methods differ in the origin, proportion, and fidelity of microglia within the organoids, and may have produced inconsistent results. To help researchers to develop a robust and reproducible model that recapitulates in-vivo signatures of human microglia to study brain development and pathology, this review summarized the current methods used to generate MC-HBOs and provided opinions on the use of MC-HBOs for disease modeling and functional studies.

## Microglia play crucial roles in the brain

**Fig. 1:**
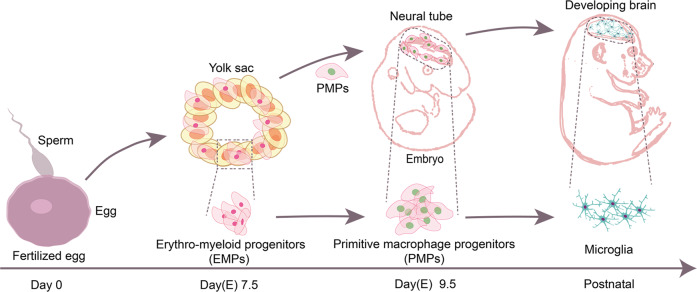
Brief developmental paths of microglia in mouse. EMPs arise at embryonic day (E) 7.5 during the first wave of hematopoiesis in the yolk sac. EMPs-derived PMPs colonize the developing brain at E9.5 and further differentiate into microglia.

Microglia are main neuroimmune cells in the brain, accounting for approximately 5–15% of the total brain cells, with heterogeneity in cell densities and transcriptional patterns across distinct brain regions [1–4]. As originating from the mesoderm lineage, microglia are derived from erythro-myeloid progenitors (EMPs) in the developing embryonic yolk sac [1, 5, 6]. The EMPs differentiate into primitive macrophage progenitors (PMPs) that migrate into the developing brain where they differentiate into microglia [5, 7] (Fig. 1). After residing in the brain, microglia are maintained through a self-renewal process [8] depending on cytokines, including interleukin (IL)-34 and colony-stimulating factor (CSF)-1 [9–11], and on transcription factors such as PU.1 and interferon regulatory factor 8 [12].

Microglia are well known for their critical functions in immune surveillance. In the brain, microglia serve as resident phagocytes that dynamically monitor the environment. In normal physiological conditions, resting microglia are highly thin and ramified. While in disease status or other conditions that disrupt the brain homeostasis or immune milieu, microglia become activated with short and thick morphologies [13]. Activated microglia rapidly respond to endogenous or exogenous pathological insults, and eliminate those pathogenic species through pinocytosis, phagocytosis, or receptor-mediated endocytosis, thus protecting the brain from damages caused by the pathological insults [14].

In addition to their immune functions, microglia also play critical roles in neural development [15], synaptic formation and plasticity [16, 17], and neural network maturation [18]. Microglia selectively colonize the cortical proliferative zones and phagocytose neural precursor cells (NPCs) to regulate neural development [19]. During postnatal brain development, microglia actively engulf synaptic material and eliminate weak synapses to shape neuronal circuits through synaptic pruning [18].

## Models to study microglia

Studies conducted over the past decades have implicated microglia dysfunction in multiple brain disorders [20], ranging from psychiatric disorders (e.g., schizophrenia, bipolar disorder, and autism) [21, 22] to neurodegenerative diseases including Alzheimer’s disease (AD) and Parkinson’s disease (PD) [23, 24]. Developing therapeutics by targeting microglia has emerged as a promising approach to the therapy of microglia-related brain disorders. To illuminate how microglia contribute to brain development and pathology, multiple microglial models including in-vivo animal models, two-dimensional (2D) cell cultures, human microglia-mouse chimeric models, and the emerging three-dimensional (3D) cultured microglia-containing human brain organoids (MC-HBOs) have been constructed. The major pros and cons of these microglial models are shown in Table 1.

**Table 1:** Major pros and cons of microglial models.

Microglial models	Sources of microglia	Advantages	Disadvantages
In-vivo animal models	1. Leech 2. Zebrafish 3. Rodents 4. Non-human primates	1. In-vivo animal brain microenvironment 2. In-vivo studies 3. Behavioral studies	1. No human genetic background 2. Long experimental period 3. Time-consuming, high-cost
2D cell cultures	1. Animal or human primary microglia 2. Animal or human microglial cell lines 3. Animal or human induced microglia-like cells	1. High cell proliferation rate 2. Short experimental period 3. Low cost	1. Limited access to human primary microglia 2. Difficult to capture in-vivo signatures of microglia 3. Microglia-neuron interactions are usually unavailable
Human microglia-mouse chimeric model	1. Human induced microglia-like cells 2. hiPSCs/hESCs-derived microglial progenitor cells	1. In-vivo mouse brain microenvironment 2. In-vivo studies 3. Behavioral studies	1. Lack interactions between microglia and other human brain cell types
3D microglia-containing human brain organoids	1. Human primary microglia 2. Human microglial cell lines 3. Human induced microglia-like cells 4. hiPSCs/hESCs-derived microglial progenitor cells 5. Spontaneous differentiation from hiPSCs/hESCs	1. Human genetic background 2. In-vivo like human brain microenvironment 3. Microglia-neuron interactions	1. Could not fully recapitulate human in-vivo brain microenvironment 2. Difficult to model adult human brain 3. Time-consuming, high-cost

*hiPSCs* human induced pluripotent stem cells, *hESCs* human embryonic stem cells.

### In-vivo animal models

In-vivo animal models including leech, zebrafish, rodent, and non-human primates have been intensively used for microglia study [25]. By the use of technologies including in-vivo labeling, living imaging, gene editing, and high-throughput sequencing, etc., animal models have provided us with much understanding of the basic characteristics, developmental paths, and functions of microglia [26]. Animal models are undoubtedly useful in uncovering important new findings of microglia biology. However, key features such as transcriptional and pharmacological differences between animal and human microglia limited the application of animal models in human microglia studies [27–30]. For example, human and mouse microglia have opposite responses to valproic acid, a drug for epilepsy and bipolar disorder. When exposed to valproic acid, mouse microglia were selectively killed but human microglia were not [29]. Another key limitation of animal models is the genetic differences between animals and humans. For example, the schizophrenia risk gene complement component 4 (C4) [31] has C4A and C4B isotypes in human, but solely C4b in mouse. Considering that greater C4A expression in the brain is associated with increased schizophrenia risk [31], the C4b knockout mouse may not be a good model for schizophrenia and other microglia pruning-related disorders. In addition, animal models usually need a long experimental period and motivate the development of in-vitro cell culture models for human microglia study.

### 2D microglial cultures

#### Primary microglial cultures

Since primarily isolated microglia have the same genetic background and similar phenotypes as in-vivo cells, microglia isolated from brains of rodents, non-human primates, and humans have been used for ex-vivo cultures [26, 32]. Especially, human primary microglia could be isolated from postmortem brain tissues, epilepsy surgery, or brain tumors [26, 32], allowing investigations to be conducted under human genetic background. However, the transfer of human or animal microglia to the in-vitro environment resulted in transcriptome-wide gene expression changes, including the downregulation of microglia-specific genes [28, 33]. The acutely isolated primary microglia are likely ex-vivo activated and may present characteristics different from the in-vivo microglia. The isolated primary microglia may also show inconsistent phenotypes since they could be isolated from different brain regions or individuals with different healthy conditions [26]. Moreover, high-quality human postmortem brains are limited to obtain, and extensive preparations are needed for each surgical operation. These limitations hinder the application of primary microglia cultures [34].

#### Microglial cell lines

Microglial cell lines are generally homogeneous cell populations with high cell proliferation rates. The use of microglial cell lines could shorten the experimental period and enable low-cost cell culture, making them suitable for basic research and high-throughput screening assays. With these advantages, a number of immortalized microglial cell lines with mouse, rat, rhesus macaque, and human origin, including the commonly used BV2, N9, and HMO6 cell lines, have been used for microglia studies [35]. However, complex cellular interactions and in-vivo like environment are absent from microglial cell lines. Studies underlined the limitations of microglial cell lines in recapitulating transcriptional signatures, morphologies, and functions of in-vivo microglia [32, 35]. Moreover, the phenotypes and transcriptional patterns of microglia may be altered after viral infection or immortalization.

#### Induced microglia-like cells (iMGs)

In addition to primary microglia and immortalized microglial cell lines, iMGs induced from mouse embryonic stem cells [36, 37] or human induced pluripotent stem cells (hiPSCs) [38–45] could be feasible models for microglia study. hiPSCs are particularly useful for their abilities to retain the donors’ genetic backgrounds, self-renew, and be amenable to gene editing. Though hiPSCs technology developed rapidly since its inception in 2006 [46], it was not until 2016 that Muffat et al. published the first protocol to generate iMGs from hiPSCs [38]. Following that, a few studies published similar strategies to generate iMGs from hiPSCs [39–45]. Detailed similarities and differences across these differentiation protocols have been well-reviewed in previous reviews [32, 47]. Here, we emphasize that iMGs generated from all these protocols functionally resemble in-vivo microglia regarding their abilities to phagocytose exogenous substances and respond to immune stimulation. Transcriptomic comparison and principal component analyses confirmed the iMGs’ resemblance to the human fetal [38–44] and even adult [38–40, 43] primary microglia.

Another strategy to generate iMGs is to transdifferentiate human peripheral blood mononuclear cells [48, 49] or hiPSCs-derived monocytes [50] into microglia by adding IL34 and GM-CSF or M-CSF into the differentiation medium. After about two weeks of culture, cells would differentiate into iMGs that transcriptionally resembled human primary microglia and showed typical characteristics of in-vivo microglia. These transdifferentiation approaches could be rapidly established and are easy to operate as an alternative iMGs model.

Though iMGs are readily available sources for microglia study, shortcomings of iMGs also exist. iMGs showed transcriptional and morphological heterogeneity across differentiation protocols. For example, iMGs generated from studies of Pandya et al. [40] and Muffat et al. [38] did not separate well from blood dendritic cells and macrophages according to the correlation-based hierarchical clustering analysis. iMGs generated in some studies [40, 45, 50] did not show highly ramified morphologies as reported in other iMGs studies [38, 39, 41–44]. Moreover, how much iMGs could mimic the in-vivo microglia are unclear since microglia-neuron interactions and in-vivo like brain microenvironment are usually absent from the iMGs culture systems.

### Human microglia-mouse chimeric model

To study human microglia in-vivo, chimeric models that transplanted hiPSCs-derived microglial progenitor cells (MPCs) [51, 52] or microglia [53, 54] into mouse brain have been developed. The xenotransplanted microglia exhibited highly ramified morphologies at a homeostatic state, recapitulated heterogeneity of adult human microglia [51, 52], and transcriptionally resembled ex-vivo cultured microglia derived from human brain tissues (age from 13 to 102 years) [51–54]. The xenotransplanted human microglia showed a differentially transcriptional response to amyloid-β (Aβ)-plaques when compared with mouse microglia, revealing new human-specific Aβ-responsive genes [53, 54]. By comparing transcriptome data from 2D-cultured iMGs [39] and iMGs transplanted into mouse brain [52], Popova et al. [55] showed that in-vivo mouse brain microenvironment significantly promoted the fidelity of iMGs. The chimeric models may better model human microglia characteristics than other in-vitro approaches [53], but several important differences between xenotransplanted iMGs and human primary microglia do exist. For example, cytokine and chemokine signatures that specifically associated with the human brain environment were significantly downregulated in iMGs transplanted into the mouse brain [55]. These findings suggested potential effects of intercellular interactions in shaping transcriptional and functional differences between human and mouse microglia [55]. A human cell-based model that contains complex cellular interactions and in-vivo like physiological conditions could be an exciting alternative for microglia study.

### 3D cultured MC-HBOs

#### Introduction of HBOs

Microglia function through interacting with other brain cell types. Intercellular signaling pathways enable microglia to communicate with one another and their surrounding microenvironment [56, 57]. Since complex cellular architectures similar to the in-vivo human brain are usually not available in 2D cell cultures, studies underscored the shortcomings of 2D microglial cultures in recapitulating in-vivo signatures [32, 55, 58, 59]. Though animal models or chimeric models could provide an in-vivo microenvironment for microglia, the lack of human genetic background or interactions between microglia and other human brain cell types is another concern for microglia study. A model containing multiple human cell types that facilitates microglia-neuron/macroglia interactions is desirable.

The 3D cultured HBOs developed in recent years become an effective solution for microglia study. HBOs are 3D self-assembled structures derived from hiPSCs or human embryonic stem cells (hESCs) that resemble the cellular organization and developmental trajectories of the human brain. In general, HBOs contain progenitors (NPCs, radial glia, etc), neuronal and glial cell types (astrocytes and oligodendrocytes), as well as other cell types [60–64]. HBOs recapitulate epigenetic and transcriptomic signatures of the fetal human brain [65, 66] and even match early postnatal brain maturation [65, 67], providing a unique platform to model human brain development and diseases. For example, Pérez et al. used HBOs to investigate functional impacts following the knockout of pitrilysin metallopeptidase 1 (PITRM1). They identified that PITRM1-knockout HBOs spontaneously developed AD pathological features, including the accumulation of amyloid precursor protein (APP) aggregates, tau pathology, and neuronal cell death. Though APP accumulation and an increased Aβ42/Aβ40 ratio were observed, neither overt cell death nor Aβ aggregates or tau pathology were detected in the hiPSCs-derived 2D neural cultures [68]. These findings highlighted the advantages of 3D HBOs against 2D cell cultures in modeling disease pathological features. Additionally, HBOs allow investigations to be conducted under human genetic background, making it attractive for disease modeling, gene function study, and drug screening. To date, HBOs have been extensively used to model a wide range of brain disorders including schizophrenia [69], bipolar disorder [70], autism spectrum disorder [71], and neurodegenerative diseases such as PD [72] and AD [73].

#### Generation of MC-HBOs

The neuronal lineage cells are of ectoderm origin. Differentiation towards endoderm and mesoderm lineages is generally suppressed during the formation of HBOs. Therefore, microglia are generally assumed to be absent from HBOs due to their non-neuroectodermal origin. Several strategies now have been developed to generate MC-HBOs to enable a better understanding of microglial functions and their connections to brain disorders. The major pros and cons of these strategies are listed in Table 2 to guide the choice for microglia study.

**Table 2:** Summary of strategies used to generate MC-HBOs.

Methods	Sources of microglia	Advantages	Disadvantages	Major applications
Co-culture of microglia with HBOs	1. Human primary microglia 2. Human microglial cell lines 3. Human iMGs	1. Abundant sources of microglia 2. Available for gene editing in microglia alone	1. Uncontrollable proportion and distribution of microglia in HBOs 2. Heterogeneity of iMGs	1. Study how microglia affect functions of brain organoids 2. Study microglia-specific genes or genetic variations
Co-culture of MPCs with HBOs	1. hiPSCs/hESCs-derived MPCs	1. Available for gene editing in microglia alone 2. Mimic the developmental trajectories of in-vivo microglia	1. Uncontrollable proportion and distribution of microglia in HBOs	1. Study how brain organoid environment affects microglia development 2. Study microglia-specific genes or genetic variations
Co-culture of MPCs with NPCs	1. hiPSCs/hESCs-derived MPCs	1. Available to study co-development of microglia and neurons 2. Controllable proportion of microglia in HBOs 3. Available for gene editing in microglia alone	1. Variable abilities of NPCs to form brain organoids	1. Study how microglia affect early neural development 2. Study microglia-specific genes or genetic variations
Spontaneous formation of microglia in HBOs	1. Innately developed within HBOs	1. Simple protocol 2. Interactions between microglia and other cell types exist throughout organoid development	1. Uncontrollable proportion and distribution of microglia in HBOs 2. Gene editing in microglia alone is not available	1. Large-sample studies

#### Co-culture of microglia with HBOs

**Fig. 2:**
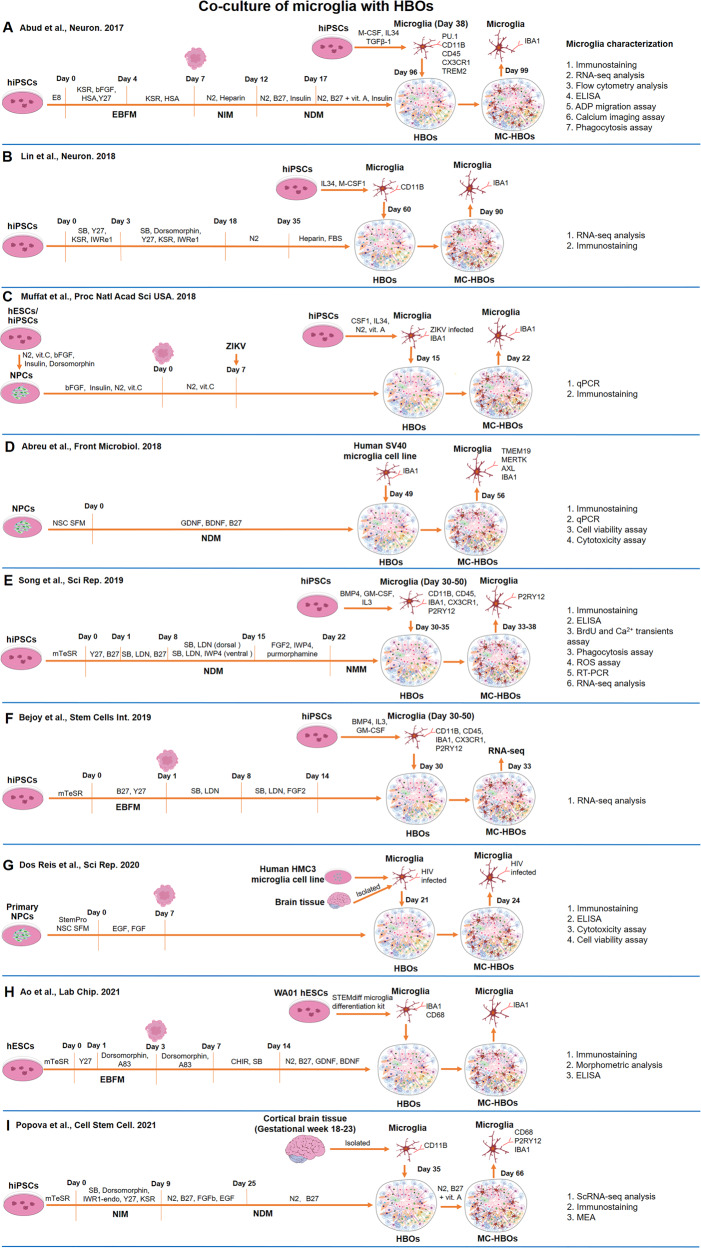
Co-culture of microglia with HBOs to generate MC-HBOs. (**A**–**I**) The sources of microglia, timeline, and key molecules used to generate MC-HBOs are presented. The markers and methods used for microglia characterization are shown. BDNF Brain-derived neurotrophic factor, CHIR CHIR99021, CSF Colony-stimulating factor, EB Embryonic body, EBFM Embryonic body formation media, EGF Epidermal growth factor, FBS Fetal bovine serum, FGF Fibroblast growth factor, GDNF Glial cell-derived neurotrophic factor, GM-CSF Granulocyte-macrophage colony-stimulating factor, HSA Human serum albumin, KSR Knockout serum replacement, LDN LDN193189, NDM Neural differentiation media, NIM Neural induction media, NMM Neural maturation/maintenance media, SB SB431542, SCF Stem cell factor, Vit.A Vitamin A, Vit.C Vitamin C, Y27 Y27632, ZIKV Zika virus.

Since microglia are thought to be absent from HBOs, we would naturally propose to co-culture HBOs with exogenous microglia to build MC-HBOs (Fig. 2). To our knowledge, Abud et al. [39] were the first to co-culture hiPSCs-derived iMGs with HBOs that contained neurons, astrocytes, and oligodendrocytes. They found that iMGs were able to integrate into HBOs, mature, ramify, and respond to injury similar as in-vivo microglia. Both iMGs in 2D culture and xenotransplanted in immune-deficient AD mice could internalize and phagocytose Aβ plaques or tau neurofibrillary tangles, two hallmark AD pathologies [39]. However, whether iMGs integrated within HBOs could phagocytose the pathological species was not investigated.

After the study by Abud et al., several other studies also incorporated iMGs into HBOs to build MC-HBOs. By co-culturing HBOs with isogenic iMGs, Song et al. [74] generated dorsal or ventral forebrain spheroids containing iMGs (D-iMGs and V-iMGs, respectively). In their study, D-iMGs and V-iMGs showed differential microglia migration ability, intracellular Ca^2+^ signaling, and the response to pro-inflammatory stimuli. Increased cell proliferation (higher BrdU^+^ cells) and reduced reactive oxygen species in D-iMGs and V-iMGs were observed when compared with those in iMGs only. By comparing bulk transcriptome from D-iMGs and from iMGs only, they identified sets of differentially expressed genes, including the neuron and glial cell-specific genes. However, it is questionable to make such a comparison since the D-iMGs contain diverse cell types in addition to microglia, while the iMGs contain microglia only. The sources of the phenotypic and transcriptional differences cannot be defined. These differences may just reflect the intrinsic cell composition differences between cortical spheroids and iMGs. Similarly, the study by Bejoy et al. [75] investigating the impact of 3D culturing microenvironment on the metabolic phenotypes of iMGs came across the same problem. They re-analyzed the transcriptome data of Song et al. [74] to identify transcriptional differences in metabolic pathways between D-iMGs and iMGs only. Genes related to glycolysis, hypoxia signaling, and several metabolic pathways were found to be upregulated in D-iMGs. However, it might be premature to conclude that 3D microenvironment may reshape the immunity of in-vitro cortical spheroids [75] by comparing transcriptome from D-iMGs and from iMGs only.

Ao et al. [76] used a stereolithography 3D printer to develop a tubular device for brain organoid culture. They stated that the tubular device reduced organoid necrosis and hypoxia. Isogenic microglia could incorporate into the tubular organoids and show a stronger cytokine response compared to that in 2D iMGs. Specifically, the level of tumor necrosis factor α (TNFα) was significantly elevated in microglia-incorporated tubular organoids but not in 2D iMGs post lipopolysaccharide (LPS) exposure, suggesting the advantage of MC-HBOs in modeling neuroinflammation.

The above studies established the MC-HBOs models, but how microglia within HBOs resemble human primary microglia and how they affect functions of HBOs were not investigated. Recently, Popova et al. [55] comprehensively compared the transcriptomic signatures of microglia across different models, including iMGs, iMGs transplanted in mouse brain, primary microglia cultured ex-vivo or transplanted in HBOs, to create a microglia report card. They found that human primary microglia transplanted into HBOs had the closest resemblance to their in-vivo counterparts, suggesting that brain organoid microenvironment preserves the homeostatic microglia state [55]. They further found that radial glia and dividing cells in HBOs were two top cell types responding to microglia transplantation. Microglia transplanted into HBOs induced transcriptional changes, reduced cell stress, and attenuated expression of interferon response genes. Microglia also increased the synchronization and frequency of oscillatory bursts in the HBOs that facilitated maturation of neural networks [55], highlighting the microglial contribution to neural development. The study by Popova et al. [55] presented better evidence to illustrate how microglia and brain organoids affect each other.

Using MC-HBOs, studies investigated how microglia acted in brain pathology. Lin et al. [77] co-cultured *APOE3-* or *APOE4*-carrying iMGs with APP gene duplication HBOs that displayed Aβ aggregates, to investigate how microglia impacted AD pathology. They found that APP duplication organoids co-cultured with *APOE4* iMGs exhibited more extracellular Aβ aggregates than those co-cultured with *APOE3* iMGs. Moreover, *APOE4* iMGs within APP duplication organoids showed longer processes than those in *APOE3* iMGs, suggesting that iMGs with *APOE4* variant were less able to sense and respond to extracellular Aβ. These results supported that *APOE4* variant impacted AD pathology mainly through affecting the ability of iMGs to clear extracellular Aβ.

Several other studies applied MC-HBOs to model ZIKA [78, 79], Dengue [78, 79], and HIV-1 viral infection [80]. For example, Muffat et al. [78] showed that ZIKA virus pre-infected iMGs penetrated the HBOs and spread ZIKA virus by initiating infection of adjacent cells, resulting in a stalled growth of HBOs. Abreu et al. [79] also used MC-HBOs to model ZIKA and Dengue viral infection by incorporating virus-infected human SV40 microglial cells into HBOs. MC-HBOs supported replication of the ZIKA and Dengue viruses. Increased expression of several cytokine/chemokine genes was observed in MC-HBOs after infection of ZIKA (*IL6, IL1β*, *TNF*α, and *CCL2*) and Dengue virus (*IL1β* and *CCL2*). However, due to the rapid and unlimited proliferation of immortalized SV40 microglial cells, the MC-HBOs could only maintain for 7 days after microglia incorporation. In another study, Reis et al. [80] incorporated HIV-1 infected human HMC3 microglial cells into HBOs, finding that HIV-1 infection induced inflammatory responses and the release of pro-inflammatory factors (TNFα and IL1β). Similar to Abreu et al. [79] study, the MC-HBOs could only maintain for 15 days after microglia incorporation. To enable a long-term culture of MC-HBOs, Reis et al. incorporated HIV-1 pre-infected primary human adult microglia into HBOs. They found that HIV-1 infection resulted into significant cytotoxicity, neuronal loss, and astrocyte activation in MC-HBOs, which are major hallmark features seen in the brains of HIV-1 infected individuals [80]. These studies indicated that MC-HBOs could support viral replication and trigger strong immune responses to viral infection.

Taken together, the above studies showed that microglia could incorporate into HBOs and reshape the immunity and functions of HBOs. This co-culture model could be a useful tool for modeling brain development and pathology. However, microglia from different studies could vary in their maturity and the abilities in response to immune stimuli, which may result into inconsistent results after microglia incorporation into HBOs. For example, iMGs in Abud et al. study [39] secreted higher levels of cytokines IL6, IL8, IL10, and TNFα after exposure to LPS. However, no change of TNFα was detected in iMGs after exposure to LPS in AO et al. study [76]. In Abreu et al. study [79], gene expression of *IL6*, *IL8*, and *IL10* was also not increased in SV40 microglial cells after LPS treatment. Interestingly, LPS treatment upregulated gene expression of *IL6*, *IL8*, and *IL10* in HBOs containing SV40 microglia when compared with HBOs without microglia [79]. These results suggest the cross-talk between microglia and other cell types including astrocytes and oligodendrocytes within brain organoids. Such cross-talk may play important roles in immune response [81, 82] and is required for a specific inflammatory response.

#### Co-culture of MPCs with HBOs

**Fig. 3:**
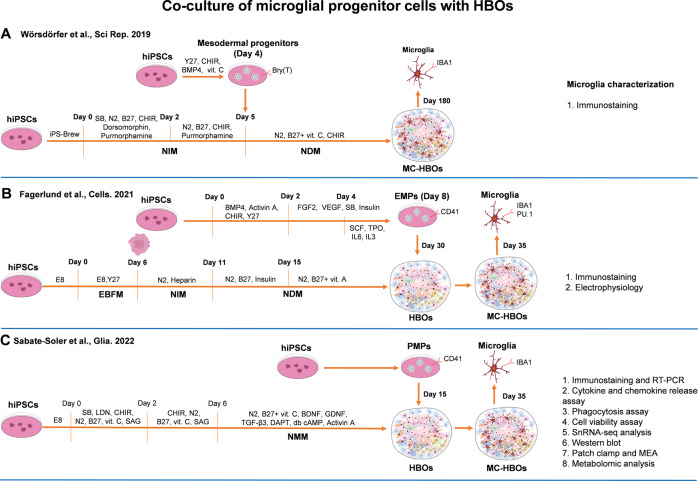
Co-culture of MPCs with HBOs to generate MC-HBOs. (**A**–**C**) MPCs derived from hiPSCs were co-cultured with HBOs to generate microglia in HBOs. EMP Erythro-myeloid progenitor, MEA multi-electrode array, SAG Smoothened agonist, TPO thrombopoietin, VEGF Vascular endothelial growth factor.

Microglia are cells of mesodermal origin. Incorporation of MPCs (EMPs or PMPs) into HBOs may also generate microglia under the microenvironment of HBOs (Fig. 3). By co-culture of hiPSCs-derived mesodermal progenitors (Brachyury^+^) with neural spheroids, Wörsdörfer et al. [83] reproducibly generated vascularized neural organoids with vessel-like structures (CD31^+^) and microglia-like cells included. This study provided a model for studying angiogenesis and neural development, but how microglia functioned in the organoids was not investigated. In another study, Fagerlund et al. [84] reported that hiPSCs-derived EMPs (CD41^+^) migrated into HBOs, differentiated into microglia-like cells, and interacted with synaptic materials. Whole-cell patch-clamp and multi-electrode array recordings showed that microglia within the organoids promoted neural network maturation. Specifically, spontaneous and NMDA-induced neuronal bursting activity was stronger and more prevalent in organoids with microglia than that in organoids without microglia. Excitatory post-synaptic currents (EPSCs) were present only in neurons from microglia- containing organoids after day 107 [84]. A recent study [85] that co-cultured human midbrain organoids with hiPSCs-derived macrophage progenitors also reported that microglia integration led to increased neural maturation and functionality. Whole-cell patch-clamp and multi-electrode array recordings showed that a lower threshold for action potential generation and a shorter interspike interval were observed in microglia-integrated midbrain organoids, suggesting that microglia integration promoted neural maturation. The presence of microglia in midbrain organoids also increased cytokine release (including IL6, IL10, IL1β, and TNFα), and affected expression of genes related to immune response, inflammation, phagocytosis, and synaptic remodeling and maturation [85].

The incorporation of MPCs into HBOs provides a promising model for microglia study, since this strategy could generate MC-HBOs with vessel-like structures included. The emergence of vessel-like structures has the possibility to improve survival and maturity of brain organoids, though the current vascular structures are far from functional. Importantly, microglia developed in this strategy mimic the developmental trajectories of in-vivo microglia, resembling the process that microglial progenitors migrate into early developing brain for further maturation. However, the amount of microglia and other potential mesodermal lineage cells developed in HBOs is uncontrolled after incorporation of MPCs. For example, the number of microglia developed in MC-HBOs using this strategy only accounted for 1.18% of total cells as revealed by single-cell RNA sequencing (scRNA-seq) [85]. This number is much lower than that in the healthy human brain.

#### Co-culture of MPCs with NPCs

**Fig. 4:**
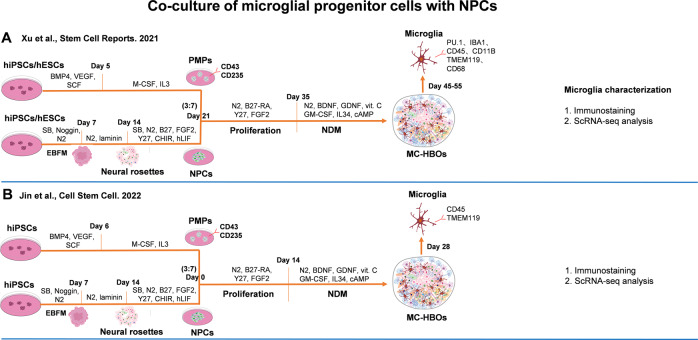
Co-culture of MPCs with NPCs to generate MC-HBOs. (**A**–**B**) MPCs derived from hiPSCs or hESCs were co-cultured with NPCs to generate MC-HBOs. hLIF human leukemia inhibitory factor, RA Retinoic acid.

Studies have demonstrated the capacity of NPCs to self-assemble into 3D brain organoids [86]. Co-culture of human MPCs with NPCs could be an alternative to develop MC-HBOs (Fig. 4). This strategy allows researchers to study the interactions between microglia and NPCs or developing neurons during the organoid formation, and to generate uniformed and cell-type ratio-controlled MC-HBOs. By co-clustering of hiPSCs-derived PMPs (CD43^+^ and CD235^+^) and NPCs, Xu et al. [87] developed brain-region specific MC-HBOs with a controllable proportion of microglia (8% in total cells). In the MC-HBOs, microglia exhibited the capacity to phagocytose NPCs, remove apoptotic cells, prune synapses, and respond to immune stimuli. The microglia could also respond to ZIKA virus and exhibit increased synaptic elimination in MC-HBOs. Importantly, neurons in the MC-HBOs (~90 days) exhibited EPSCs, suggesting the establishment of functional neural network in the organoids.

By using Xu’s co-culture methods [87], Jin et al. [88] generated MC-HBOs to explore microglial functions in Down syndrome (DS). In their study, DS microglia in HBOs exhibited enhanced synaptic pruning without significantly morphological changes when compared with control microglia. After transplantation into mouse brain, both DS and control microglia showed increasingly complex morphologies. However, the xenografted DS microglia displayed less intricate morphologies than control microglia, as indicated by enlarged cell volume, shortened process length, fewer endpoints, and branch numbers. These results suggested that MC-HBOs recapitulated the enhanced synaptic pruning function of DS microglia but were not optimal for modeling the morphological changes of DS microglia. This might be due to that microglia in HBOs did not exhibit highly branched and ramified morphologies as they did in-vivo. In addition, the MC-HBOs were limited to examine how DS microglia affected synaptic transmission in HBOs, as neurons didn’t exhibit robust EPSCs until after a long-term culture (~90 days) [88]. The authors transplanted microglia into mouse brain and found that DS chimeric mice had miniature EPSCs with significantly reduced frequency and amplitude. DS microglia showed dystrophic and senescent phenotypes in response to pathological tau. Reducing expression of Hsa21 encoded type I interferon receptor genes (IFNARs) rescued the accelerated senescence and dystrophic phenotypes of DS microglia [88], implying therapeutic targets for treating AD in DS.

#### Spontaneous formation of microglia in HBOs

**Fig. 5:**
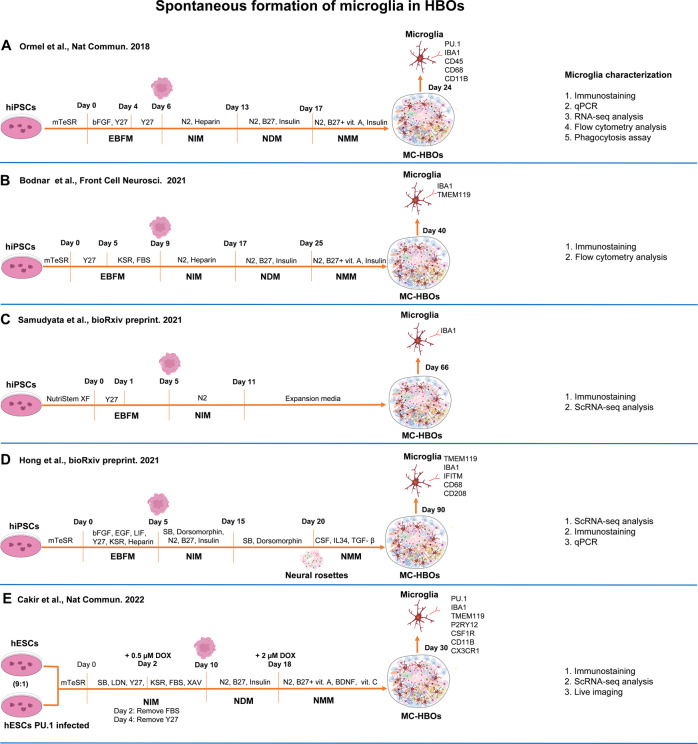
Spontaneous formation of microglia within MC-HBOs. (**A**–**E**) hiPSCs or hESCs were used to generate microglia that innately developed within the brain organoids. XAV XAV939, DOX Doxycycline.

Pluripotent stem cells including hiPSCs and hESCs have the potential to differentiate into all cell types of three germ layers (endoderm, mesoderm, and ectoderm). Due to the ectoderm origin of neuronal cells, dual-SMAD signaling inhibition was commonly used for neuroectoderm induction in order to suppress the differentiation towards endoderm and mesoderm lineages [89]. Since SMAD signaling inhibitors were not used in the unguided protocol for cerebral organoid formation [60], it is possible that non-neuronal lineage cells exit in the cerebral organoids. Indeed, scRNA-seq analysis identified mesodermal progenitors [62], myeloid cells [90], and even microglia clusters [68] in HBOs generated from unguided protocols. With the hypothesis that mesodermal progenitors within HBOs are able to differentiate into mature microglia under the organoid microenvironment, Ormel et al. firstly developed a model with microglia innately developed within cerebral organoids [91] (Fig. 5). Interestingly, the authors only simply modified the unguided protocol by reducing the concentration of heparin (from 1 ug/mL to 0.1 ug/mL) and delaying matrigel embedment of the organoids, resulting in the production of mesodermal progenitors and following microglia generation in organoids (as early as day 24). The organoid-grown microglia displayed ramified morphologies, expressed microglia-specific markers, functionally resembled adult human microglia, and elicited immune responses in organoids. Interestingly, EPSCs could be detected in MC-HBOs as early as day 52 in Ormel et al. study [91], while it was not until organoid day 90 and day 107 that EPSCs could be detected in the studies of Xu et al. [87] and Fagerlund et al. [84].

Based on the protocol of Ormel et al. [91], other studies [92–94] also generated HBOs containing innately-developed microglia. The MC-HBOs were used to model SARS-CoV-2 infection [93] or Western Pacific Amyotrophic Lateral Sclerosis and Parkinsonism–Dementia Complex (ALS-PDC, a neurodegenerative disease) [94]. Upon SARS-CoV-2 infection, the innately-developed microglia in organoids responded to viral infection through upregulating phagocytosis- and interferon-related pathways. Several AD and multiple sclerosis susceptibility genes were also found to be upregulated in microglia upon SARS-CoV-2 infection, suggesting potential contribution of SARS-CoV-2 to neurological symptoms [93]. By using MC-HBOs, the ALS-PDC study [94] revealed that the shift of microglia to a more pro-inflammatory state, which exacerbated inflammation and reduced extracellular matrix strength, may account for the etiology of ALS-PDC. Unlike previous studies [91–93], this study didn’t reduce the concentration of heparin to 0.1 ug/mL but also resulted into microglia generation in cerebral organoids.

Though microglia could innately develop in HBOs, the proportion and distribution of microglia in organoids are highly variable due to the spontaneous and stochastic features of unguided differentiation [91]. For example, the proportion of CD11B^+^ microglia in MC-HBOs was about 7% in Bodnar et al. study [92], while it was up to 1.5% in the study of Ormel et al. [91]. To develop HBOs with a tunable proportion of microglia included, Cakir et al. [95] mixed myeloid-specific transcription factor PU.1-overexpressed hESCs (10%) with parental hESCs (90%) to generate MC-HBOs. scRNA-seq analysis revealed that transcriptional profiles of PU.1-induced microglia resembled those in the middle to late stages of fetal microglia development. Notably, the authors stated that the PU.1-induced microglia demonstrated a more similar transcriptome profile to human primary microglia than microglia generated in Omel’s study [91]. The presence of microglia within HBOs significantly attenuated transcriptional dysregulation of dendritic and synaptic development and apoptotic genes caused by Aβ exposure. Using the MC-HBOs, the authors further found that suppression of AD risk genes (*TREM2* and *SORL1*) in PU.1^+^ cells did not affect microglia generation but their responses to Aβ exposure. This study provided a strategy to develop a tunable proportion (~10%) of microglia within organoids for microglia study. However, not all PU.1 overexpressed cells in organoids were converted into microglia-like cells, leading to these myeloid cells with a distinct identity and maturity in organoids [95].

## Perspectives and future directions

Microglia undoubtedly play critical roles in brain development and homeostasis. Increasing evidence has linked microglia dysfunction to various brain disorders. Studies based on genetics, human postmortem brains, clinical imaging, and animal models strongly suggested microglia as a central cell type contributing to the etiology of AD [14, 24, 96–99]. Studies also suggested microglia as a key modulator of α-synuclein toxicity in PD [100, 101]. In the mouse model of PD, activation of microglia promoted cell-to-cell transfer of α-synuclein aggregates [100], while inhibition of microglia-mediated inflammation alleviated α-synuclein pathology and dopaminergic neurodegeneration [101]. In addition to neurodegenerative diseases, microglia dysfunction also connects to a wide range of psychiatric disorders [21, 22]. Studies including genome-wide association study [31] and gene coexpression analysis based on human postmortem brain transcriptome data [102] suggested the pathological involvement of microglia in schizophrenia, bipolar disorder, and autism. A robust microglial model that mimics the in-vivo human brain is needed for understanding the roles of microglia in brain disorders.

The recently developed MC-HBOs have become a promising model to study how microglia modulate brain development and pathology. Several strategies now have been developed to generate MC-HBOs to study brain development and disorders and viral infection as reviewed in this paper. Those studies highlighted MC-HBOs in resembling in-vivo microglia signatures, such as human-specific expression patterns of cytokine and chemokine genes [55] and unique inflammatory responses to viral infection [79]. In such an emerging field, most of the current studies focused on establishing robust MC-HBOs models. Regarding future work, the incorporation of microglia into HBOs could be used to study how microglia-specific genes or genetic variations affect brain phenotypes, and how microglia per se modulate brain functions. The co-culture of MPCs with HBOs, which may better mimic the developmental trajectories of in-vivo microglia, could be utilized to study how HBOs environment affects microglia development. The co-culture of MPCs with NPCs could be used to investigate co-development of microglia and neurons. More importantly, co-culture of MPCs with NPCs [87, 88] could generate MC-HBOs with a controllable proportion of microglia (~8%) similar to the physiologic conditions. In addition to co-culture methods, the strategy that innately develops microglia within HBOs is a simple method to generate MC-HBOs for the study of genetic variations existing in all cell types. This simplified method is suitable for large-sample studies such as case-control transcriptomic studies based on MC-HBOs. Considering that microglia affect transcriptional patterns and phenotypes of brain organoids, applying MC-HBOs to case-control transcriptomic studies may yield novel insights that could be missed by studies based on brain organoids without microglia.

We have to keep in mind that a robust brain organoid model is essential to yield reproducible results for microglia studies. It is urgent to put forward gold standards to define what “true” brain organoids are, as diverse protocols will likely generate HBOs with distinct features that lead to inconsistent results. A human brain organoid may have to show similar cell composition (progenitors, neurons and glia cells, etc.), cellular structures (the emergence of ventricular zone, cortical layers, etc), gene expression patterns, and developmental trajectories as the in-vivo human brain. Thus, it is challenging to call 3D cell cultures by mixing neurons with other cell types (e.g., microglia and astrocyte) as “organoids”, since cellular organization and composition, and cell developmental paths in the cell mixture system could not mimic those in the human brain. For the reasons, those studies [103, 104] that developed 3D assembloids [105] by co-culture of neurons, microglia, and astrocytes were not included in this review. Assembloids, as defined by Pasca [105], are 3D structures formed from the fusion and functional integration of multiple cell types. MC-HBOs formed by the co-culture strategies could also be defined as assembloids [105]. In the future, generating assembloids by fusing MC-HBOs with different brain region identities will be beneficial to study regional heterogeneity of microglia.

In addition to the standards for brain organoids, the fidelity of microglia within the MC-HBOs is another key concern in microglia study. Microglia is a highly complex and dynamic cell type. The morphologies and transcriptional patterns of microglia could be changed in response to environment. How much the induced microglia can mimic in-vivo cells to produce consistent responses remains to be learned. Microglia produced by different protocols could vary in their origin and the abilities to recapitulate in-vivo signatures. For example, hiPSCs-derived microglia from different methods showed transcriptional and morphological heterogeneity, resulting in potential heterogeneous phenotypes after incorporation into HBOs. Assays used to examine microglia identity and state, phagocytotic function, response to immune stimuli, etc., are required for microglia characterization. A systematic evaluation is also needed to assess which strategy is better to generate MC-HBOs that recapitulate the true ontogeny and in-vivo signatures of human microglia.

This review highlights the merits and applications of MC-HBOs among the several microglial models, some apparent shortcomings of current MC-HBOs should be noted. First, MC-HBOs are in-vitro models in nature and unlikely fully recapitulate features of in-vivo human brain. Current MC-HBOs lack the vascular systems and lead to increased cellular stress that hinders cell type specification and maturation of brain organoids [106]. Moreover, the lack of complex neural circuits and intact immune system also limit the further maturation of HBOs to study adult brain and late-onset brain disorders using current MC-HBOs. In the future, approaches including air-liquid-interphase culture [107], vascularization system [108], and microfluidic system [109] could be applied to brain organoid culture to attenuate cellular stress, and to promote organoid maturation.

Every microglial model has its advantages and disadvantages (Table 1), using the appropriate model in a specific situation or combining the use of different models could yield robust results for microglia study. For example, researchers combined the use of HBOs and mouse model and found that HBOs established long-distance subcortical projections in the mouse brain after transplantation. Neurons differentiated from the transplanted HBOs functionally integrated into mouse neural circuits [110, 111]. The HBOs-mouse chimeric models enable investigations to be conducted under in-vivo like conditions. Since mouse model could provide an in-vivo microenvironment to enhance fidelity of native microglia state [55], and to promote maturation of brain organoids [106, 111–113], MC-HBOs transplanted into mouse brain could be a promising model to better mimic the real human brain.

In summary, MC-HBOs have become a promising model to illustrate what roles microglia play in brain functions and disease etiologies. The MC-HBOs are not only valuable for microglia research, but also beneficial to the construction of organoids that better mimic the real human brain. With the MC-HBOs, we have the unique opportunity to advance our understanding of how the human brain develops and how it works. In the future studies, developing a robust and reproducible MC-HBOs model by combining with gene editing and multi-omics analyses will largely promote the fields of disease modeling and functional studies.
